# Analysis of Image Feature Characteristics for Automated Scoring of HER2 in Histology Slides †

**DOI:** 10.3390/jimaging5030035

**Published:** 2019-03-10

**Authors:** Ramakrishnan Mukundan

**Affiliations:** Department of Computer Science and Software Engineering, University of Canterbury, Christchurch 8140, New Zealand; mukundan@canterbury.ac.nz; Tel.: +64-3-369-2201

**Keywords:** uniform local binary patterns, characteristic curves, whole slide image processing, feature analysis, region connectivity, entropy

## Abstract

The evaluation of breast cancer grades in immunohistochemistry (IHC) slides takes into account various types of visual markers and morphological features of stained membrane regions. Digital pathology algorithms using whole slide images (WSIs) of histology slides have recently been finding several applications in such computer-assisted evaluations. Features that are directly related to biomarkers used by pathologists are generally preferred over the pixel values of entire images, even though the latter has more information content. This paper explores in detail various types of feature measurements that are suitable for the automated scoring of human epidermal growth factor receptor 2 (HER2) in histology slides. These are intensity features known as characteristic curves, texture features in the form of uniform local binary patterns (ULBPs), morphological features specifying connectivity of regions, and first-order statistical features of the overall intensity distribution. This paper considers important properties of the above features and outlines methods for reducing information redundancy, maximizing inter-class separability, and improving classification accuracy in the combined feature set. This paper also presents a detailed experimental analysis performed using the aforementioned features on a WSI dataset of IHC stained slides.

## 1. Introduction

Whole slide images (WSIs) of entire biopsy slides facilitate the processing of a wide range of features extracted from regions of interest for applications such as automated scoring of the tumour characteristics seen in the images [1]. WSIs typically contain billions of pixels at high magnifications (up to 40×) with down-sampled versions at different scales. Powerful digital scanners provide the technology to convert the information in physical slides to WSIs (also known as virtual slides) that can be processed by image analysis software for the extensive analysis of complex tissue features [2]. One of the advantages of digital pathology systems is that WSIs can be easily stored and, if required, almost instantaneously transmitted to a different location in a reliable and secure manner for processing and review by multiple pathologists. WSIs have therefore found a wide range of applications in automated computer-aided diagnosis [3].

Recent studies on medical image classification algorithms have emphasized the need for developing image analysis methods that can measure biomarker-specific features [4,5]. In the past few years, advances in WSI instrumentation have provided the ability to automatically load up to 300 slides without user intervention, with considerably faster scanning speeds [5]. This has resulted in the need for fast image processing algorithms that can detect and analyse various image and texture characteristics, and accurately extract cytological and morphological features that are relevant to histopathological studies and diagnosis. In this context, automatic image classification algorithms in the field of breast cancer diagnosis have recently received significant attention [6,7]. Some of the recently organized online contests and programming challenges also point to the need for accelerating the development of image analysis algorithms for the automated scoring and classification of breast cancer histology slides [8,9].

The overexpression of human epidermal growth factor receptor 2 (ERBB2 or HER2) protein in immunohistochemistry (IHC) stained slides is an important cell membrane biomarker used for breast cancer diagnosis [10]. Breast tissue samples are assigned HER2 scores 0, 1+ (negative), 2+ (equivocal) or 3+ (positive: aggressive disease) depending on the intensity, percentage and pattern of membrane staining observed in IHC-stained slides [11]. In order for classification algorithms to provide better accuracy and diagnostic concordance with pathologist’s assessments, it is desirable to use biomarker-specific features [12,13]. Various types of features such as intensity features, texture features and morphological features can be extracted from regions of interest within a given image. For example, features describing the intensity and completeness of membrane staining were used in [14]. Such approaches commonly use a membrane segmentation step consisting of colour-based pixel classification, nuclei identification, watershed and ellipse fitting algorithms [14,15,16]. In Reference [17], the cell regions are detected in a pre-processing step using the watershed algorithm, and then classified using deep learning into immune cells, stroma cells, tumour cells and artefacts. When there are a large number of features in an image, the information content and the discriminating power of the feature set will need to be evaluated in detail. A thorough feature analysis will help in significantly reducing information redundancy in a feature set and increasing the inter-class separability of the features [17]. A combination of multi-level features including histogram of oriented gradients, local binary patterns and Haralick features is proposed in [18].

Two important image features associated with HER2 over-expression in IHC-stained slides—namely, (i) characteristic curves [19] and (ii) rotation-invariant uniform local binary patterns (ULBPs) [20]—were recently introduced. Characteristic curves provide information about the variation of the observed percentage of staining with respect to saturation thresholds used for the stain colour, and are often represented as a one-dimensional smooth curve. When feature points fall along a smooth curve, we can make use of the information redundancy in the set to reduce the dimension of the feature vector. On the other hand, higher-order texture descriptors such as the ULBP contain a large number of feature components. However, these features possess similar geometrical characteristics and show inter-dependency in their magnitude and shape. An analysis of their parametric variations is useful for both feature reduction. Some of the common geometrical characteristics of the ULBP feature curves can be used to get a two- or three-dimensional representation of the feature vector for visualizing how the points are clustered within each class. Our previous work [21] used Fisher linear discriminant analysis (LDA) and principal component analysis (PCA) to evaluate the effectiveness of the features, reduce feature dimension, combine features of different types, maximize inter-class separability and to improve the overall classification accuracy. Such discriminant analysis approaches are commonly used to study the feature transformations used in multi-class classification algorithms [22,23]. 

In this paper, we use a different approach of analysing both geometrical and statistical properties of feature components to minimise redundancy in the set. This paper also considers the connectedness of stained membrane regions as an important feature to be used in the classification process. We define a measure of connectedness based on the size of the largest stained connected component in an image tile after thresholding using hue and saturation values. We also augment the feature set using the entropy and energy of the image after transforming it to a grey-level image in the CIE-Lab space. This paper makes contributions to the understanding of biomarker-specific features useful for HER2 classification by considering the above four types of features, namely, intensity, texture, morphological features and colour-sliced histograms, analysing their characteristics for reducing feature dimension, and demonstrating their feature representation capabilities using classification algorithms. 

This paper is organized as follows. The next section gives a description of the dataset and methods used in our analysis. Section 3 gives an overview of characteristic curves, which are features based on intensity variations. Section 4 discusses the computation of rotation-invariant uniform local binary patterns. Section 5 introduces a connectedness measure that is useful as a morphological feature for HER2 classification. Section 6 outlines first-order statistics of grey-level distribution in CIE-Lab space, useful for our analysis. This section also gives an overview of changes in classification accuracy and feature dimension at each processing stage. Section 7 gives a few geometrical characteristics of the features that could be used for dimensionality reduction. Section 8 gives a summary of the work presented in the paper, and outlines future research directions.

## 2. Materials and Methods

The dataset used in this research work consisted of a total of 172 whole-slide images in Nano-zoomer Digital Pathology (NDPI) format, corresponding to 86 cases of patients with invasive breast carcinomas [7]. WSIs of both Haematoxylin and Eosin (H&E)-stained slides and immunohistochemically-stained slides were provided for each case. The images were released to research groups by the University of Warwick as part of an online HER2 scoring contest held in 2016 [8]. The contest organizers granted permission to the participating teams to use the dataset for research purposes. For training classification algorithms, a set of ground truth data was provided. It consisted of the HER2 scores assigned for each case and also the observed percentage of membrane staining in the tissue sample as determined by expert pathologists. 

For our experimental work, we used 52 WSIs of IHC-stained images from the training dataset, with 13 WSIs belonging to each of the four HER2 classes. The H&E slides were not used in our work. Each WSI image was further subdivided into approximately 80 small tiles (image patches) of size 512 × 512 pixels for the computation of features. From this set, image tiles containing less than 40% of the region of interest (membrane regions) were removed. A total of 4019 image patches were used as samples in our classification experiments. For HER2 classification, we require features that represent the percentage and intensity of membrane staining, and the morphology of staining patterns (i.e., texture and connectedness). We therefore designed characteristic curves which efficiently represent the variation of percentage of staining with saturation levels [19,20]. The local binary patterns were used to capture local texture characteristics [20]. In this paper, we also introduce a connectedness measure that represents the connectedness of the stained membrane regions. Two global histogram features (i.e., energy and entropy) are included in the feature set, as they also showed significant variation for each class, with good inter-class separability. The features are described in detail in the following sections. As shown later in Table 1, a total of 38 features were computed per sample. For each run of the classification algorithm, this set of 4019 samples was further subdivided randomly into a training set consisting of 2813 samples (70%), and a cross-validation set consisting of 1206 samples (30%). In this paper, we show that the selected features provide a good level of accuracy using two classification algorithms: one-vs.-all logistic regression and support vector machine. The focus of the paper is on the computation and analysis of biomarker-specific features for HER2 classification, and the machine learning algorithms are used only to evaluate the feature representation capabilities of the selected set.

## 3. Characteristic Curves

**Figure 1:**
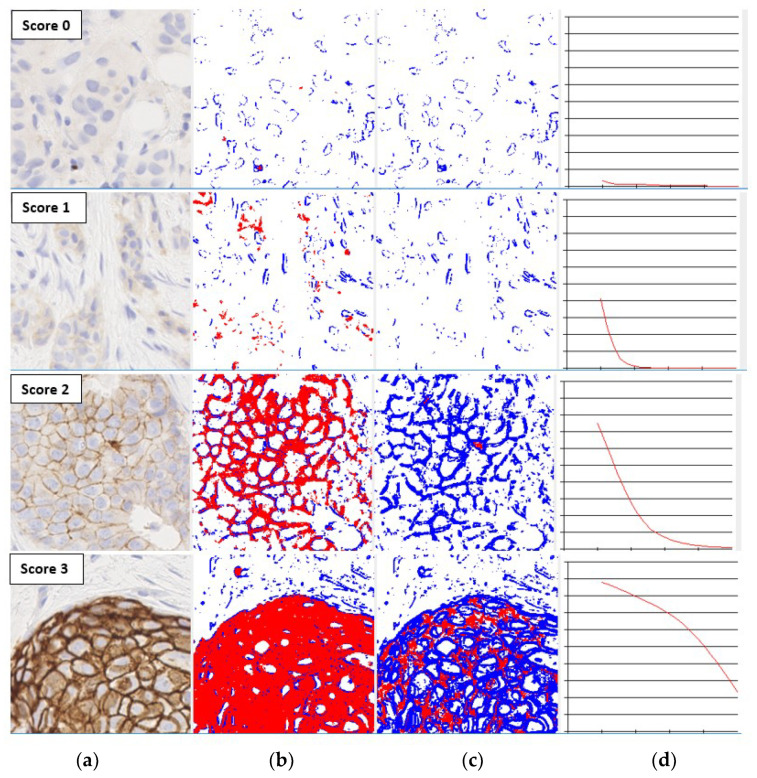
The shapes of the characteristic curves for images with different human epidermal growth factor receptor 2 (HER2) scores (**a**) Input image; (**b**) Thresholded image at saturation 0.1; (**c**) Thresholded image at saturation 0.5; (**d**) Characteristic curve with *x*-axis denoting saturation from 0 to 0.5, and *y*-axis % of stained region.

The level of membrane staining in an IHC image can be represented using a smooth curve known as the characteristic curve, which shows the variation of the percentage of pixels above a saturation threshold as the threshold value is increased within an experimentally determined range. The computational aspects of characteristic curves and their properties are detailed in [19,20]. The properties that make characteristic curves excellent candidates for intensity-based feature descriptors are their magnitude and drop-off rate, which vary significantly with HER2 scores as shown in Figure 1. The shapes of the characteristic curves can therefore be directly correlated with the staining levels required for HER2 scores as per the assessment guidelines [11]. For example, the characteristic curve always lies below the 10% threshold when the score is 0, and only a small initial segment of the curve lies above the 10% mark when the score is 1. If the score is 3+, the curve lies completely above the 30% mark, showing a strong and complete membrane staining. As seen in Figure 1, the curve passes through a much wider range of values of percentage staining when the score is 2+.

**Figure 2:**
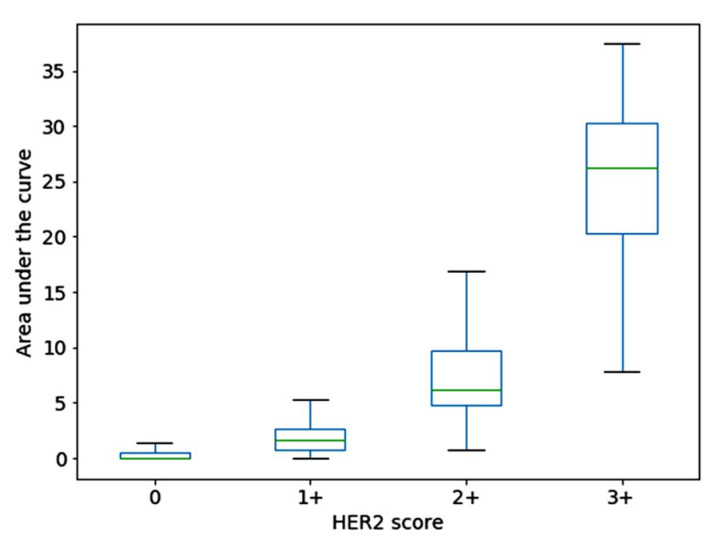
Box plot showing the distribution of the area under characteristic curves for an input dataset containing 1271 samples.

The characteristic curves used in our work have 21 points corresponding to saturation thresholds varying from 0.1 to 0.5 in steps of 0.02. Since all characteristic curves have a non-increasing trend and are defined only between pre-determined saturation thresholds along the *x*-axis, one global characteristic of the shape is the area under the characteristic curve. A box-plot showing the distribution of area in the dataset containing 1271 randomly selected samples is shown in Figure 2. The single metric itself shows a good inter-class separation of the feature vectors and can be used to visualize their distribution. 

## 4. Uniform Local Binary Patterns

**Figure 3:**
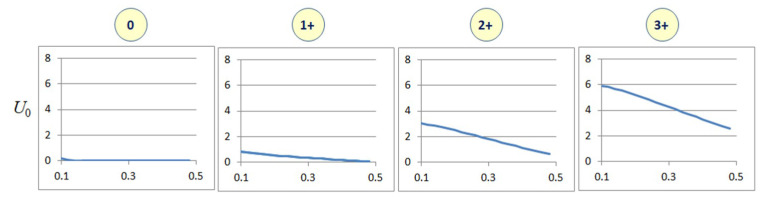
Variations of a uniform local binary pattern (ULBP) component with saturation thresholds for images with different HER2 scores. The *x*-axis represents saturation values from 0.1 to 0.5, and the *y*-axis represents the ULBP feature values.

Local binary patterns (LBPs) are texture descriptors specified using the pattern of variation of intensity values around pixel neighbourhoods [24]. Texture features based on LBPs find applications in pattern analysis, texture classification and computer vision. Since WSIs do not have any predefined orientation, the LBPs computed for WSIs must be rotation invariant. For this, we use uniform local binary patterns (ULBPs) [20]. The computation of nine ULBP components *U*_0_…*U*_8_ is detailed in [20]. We disregard U8 as it mainly represents background regions of constant intensity. Similar to characteristic curves, each ULBP feature curve also consists of 21 sampled points corresponding to variations in the saturation threshold from 0.1 to 0.5. Therefore, the whole feature vector *U*_0_…*U*_7_ has a total dimension of 168. Figure 3 shows the variation of the first three ULBP components with the saturation threshold plotted along the *x*-axis. Similar variations are seen in the remaining ULBP values. The ULBP feature curves show considerable difference in their magnitude and variance between classes with HER2 scores 1+, 2+ and 3+. However, the variance is found to be small between classes 0 and 1+ because between those two classes, there is no significant difference in the texture of staining patterns. Similarly, when the saturation threshold is increased, regions become more uniform in colour values, and hence the LBP values all tend to zero.

The ULBP feature curves generally have very low curvature and allow a first-order approximation where each curve is parameterized into the slope and the *y*-intercept of the approximating line. This linear approximation helps us to visualize their distribution in a training set.

**Figure 4:**
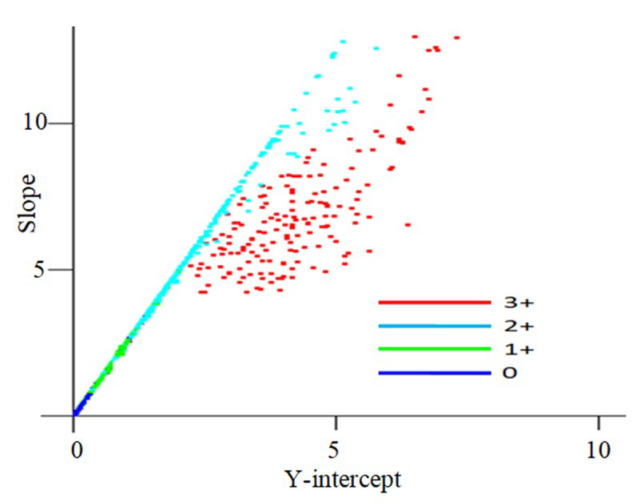
Two-dimensional parameterization of ULBP feature vectors.

Figure 4 shows the distribution of points for ULBP feature curves obtained from 900 samples. This figure clearly shows the clustering of points in each class, as well as their inter-class separation. Figure 4 also shows an important aspect of the ULBP features—they had a much wider range of variation in slopes with height value for HER2 class 3+, while for other classes, the slope varied nearly linearly with the *y*-intercept. When the intensity and percentage of staining were low, as in HER2 classes 0 to 2+, the variations in texture were nearly uniform. Significant variations in texture patterns were observed for HER2 class 3+ where the staining intensity was high.

## 5. Region Connectedness

The connectedness of stained membrane regions is also an important visual marker used by pathologists in the assessment of histology slides. Measures for connectedness have also been considered in classification algorithms [25]. The filtered region of interest (ROI) obtained from each image tile is first thresholded at the lowest saturation value (0.1) to obtain a binary image where white pixels represent stained regions. A connective component algorithm then finds the size of the largest connected component in the image. The ratio of the size to the number of pixels in the filtered region expressed as a percentage is used as the connectedness measure.

**Figure 5:**
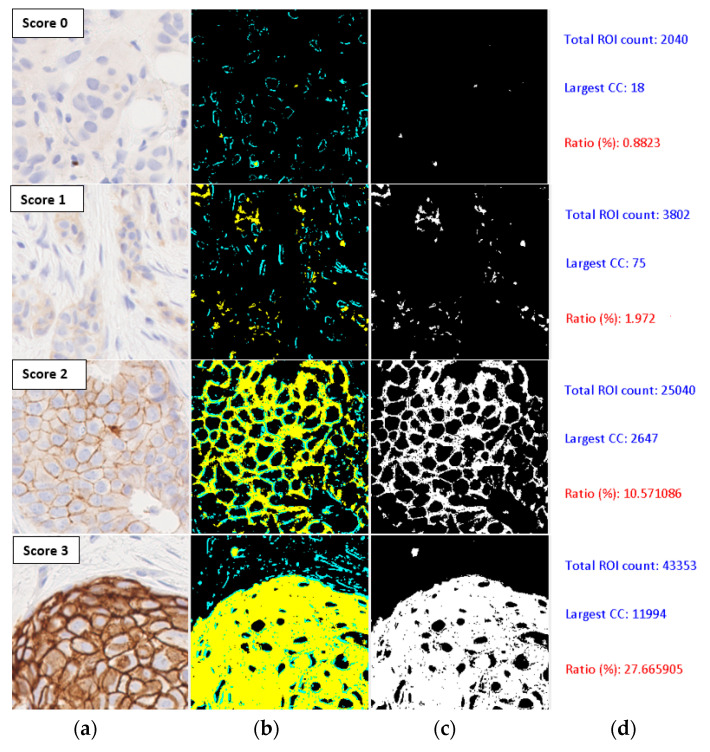
Computation of connectedness measure. (**a**) Input image; (**b**) Stained regions; (**c**) Thresholded binary image; (**d**) Computed values of connectedness parameters. ROI: region of interest, CC: connected component.

**Figure 6:**
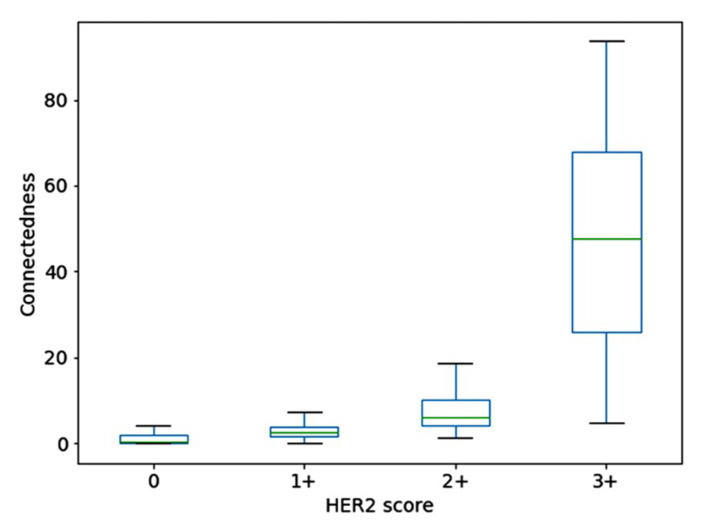
Box plots showing the distribution of connectedness value for each HER2 score.

Figure 5 shows the processed images of four tiles corresponding to four HER2 scores, with the second column showing the stained regions marked in yellow and unstained areas in the region of interest in cyan colour, for a saturation value of 0.1. The third column shows the binary image obtained by thresholding. The number of pixels in the region of interest and the largest connected component are shown in the last column. The connectedness measure is computed as the percentage ratio of the two sizes (also shown in red colour in the last column). A box-plot showing the distribution of the connectedness values in the dataset containing 1271 samples is shown in Figure 6. Even though there was a clear separation of the interquartile ranges (approx. 67%) corresponding to each score, the overall range of connectedness values overlapped between scores. 

## 6. Histogram Statistics

**Figure 7:**
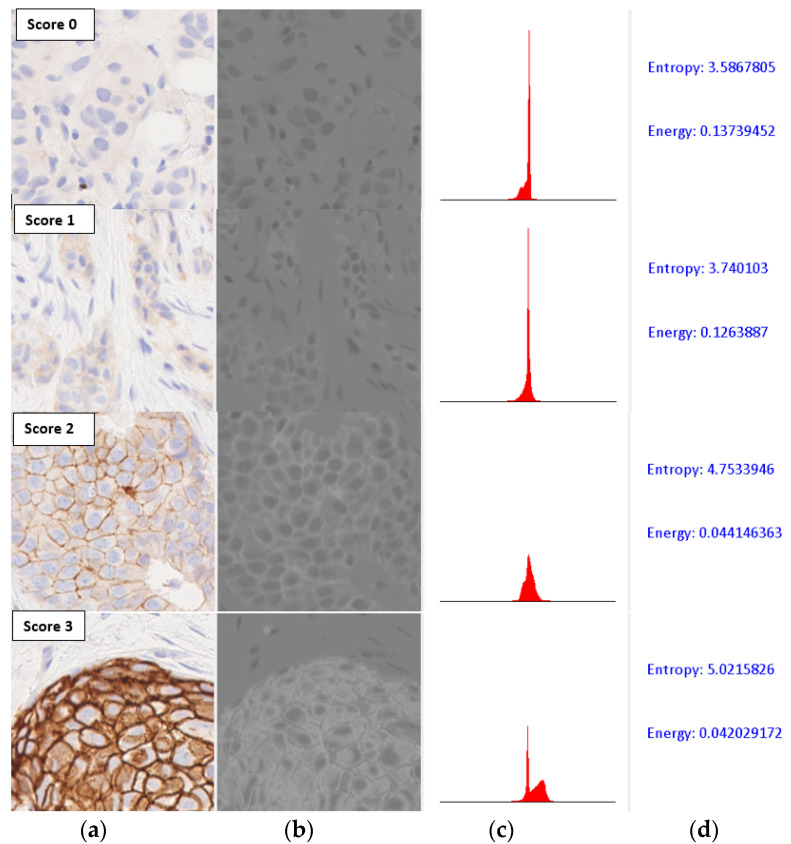
Computation of entropy and energy values. (**a**) Input image; (**b**) Grey-level image of b* channel in CIE-Lab colour space; (**c**) Histogram of the grey-level image; (**d**) Parametric values.

The distribution of colour values that are relevant for classification can be characterized by first-order statistics of the histogram of the image tile after appropriate colour-space transformation. Since most of the colour values of interest in an image of the IHC-stained slides fell along the blue–yellow axis, we first converted the input images to the CIE-Lab space [26], and used the grey-level histogram of the b* channel to compute the entropy and energy of the colour distribution (Figure 7).

**Figure 8:**
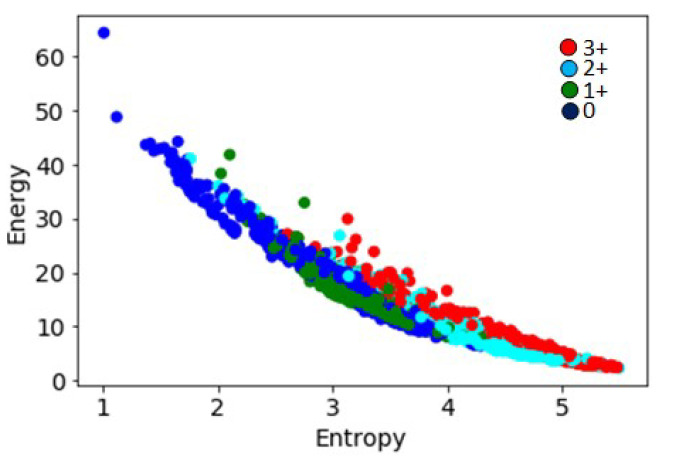
Energy and entropy values for 1271 image tiles with different HER2 scores showing an inverse correlation between the two parameters.

The entropy and energy computed from normalized histograms of grey-level images are commonly used in image classification algorithms [27]. As can be seen in Figure 7, the entropy values on the b* channel were strongly correlated with the amount of staining present in the images, and hence with the HER2 scores. However, the energy values varied inversely with increasing HER2 scores. The values plotted for all image tiles in the data set also showed an inverse correlation between the two parameters, with entropy increasing and energy reducing with increases in HER2 scores (Figure 8).

## 7. Feature Dimension

In this section, we take a look at some of the important geometrical properties of the features presented in the previous sections in order to reduce the size of the feature set to the minimum required level for classification, without transforming them to a different space. The motivation for this approach was to retain the primary visual characteristics of the features that are directly correlated with the staining patterns seen in the images. Transformation-based methods using principal component analysis (PCA) and linear discriminant analysis were presented in our previous work [21].

**Figure 9:**
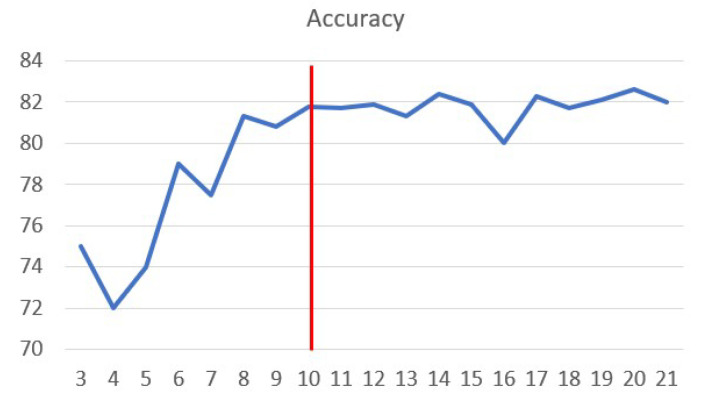
Variation of classification accuracy with the number of points on the characteristic curves.

The characteristic curves were smooth curves that could be approximated by cubic polynomial curves. However, replacing the points with polynomial coefficients affected the accuracy of classification results. We analysed accuracy variations by increasing the sampling interval on the curve to select the correct number of points for representing the features (Figure 9). Based on the experimental results, 10 feature points were selected for representing the characteristic curve. In this experiment, we used only characteristic curves as features, and the one-vs.-all logistic regression algorithm was run 200 times, randomly selecting 1206 samples from the input set for cross-validation in each run. Figure 9 shows the average accuracy from these 200 trials. 

As previously shown in Figure 3, the values of uniform local binary features *U*_0_…*U*_7_ exhibited a low curvature variation with saturation thresholds. This geometrical property was helpful in reducing the number of points on each curve from 21 to 5. Experimental results also showed similarity between several ULBP curves. A pair-wise similarity test revealed that the pairs {*U*_3_, *U*_5_}, {*U*_2_, *U*_6_} and {*U*_6_, *U*_7_} had high levels of similarity. Using this result, we could reduce the number of ULBP feature curves from 8 to 5 (*U*_0_…*U*_4_). Table 1 gives a summary of the features proposed in this paper and their dimensions based on the analysis presented above.

**Figure 10:**
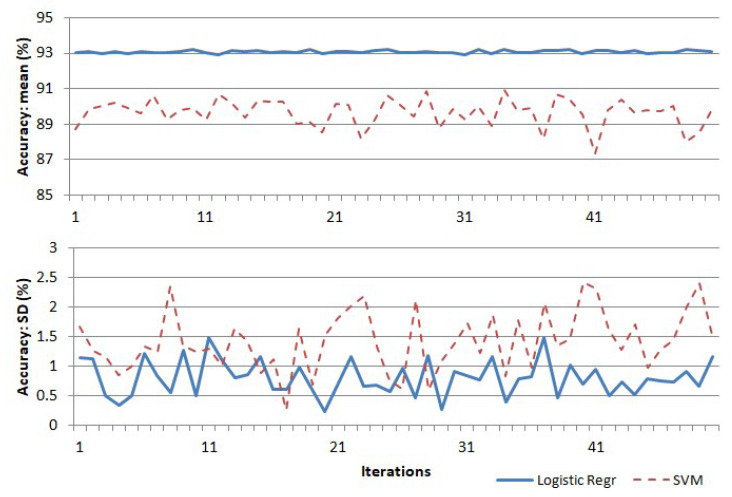
Variation of classification accuracy with repeated runs of the logistic regression and support vector machine (SVM) algorithms.

For experimental validation of the suitability of the features, the above features were used in a “one-vs.-all” multi-class classification algorithm based on logistic regression and support vector machine [28], using 5-fold cross validation. The experiment was repeated 50 times, and the plots of the mean and standard deviation of accuracy with each trial are shown in Figure 10. The logistic regression algorithm gave an average classification accuracy of approximately 93%, with a maximum standard deviation of 1.5% in accuracy. The support vector machine algorithm gave a comparatively lower score of accuracy at approximately 89% and a higher standard deviation of 2.5. A sample confusion matrix obtained from one of the runs of the logistic regression algorithm is given in Table 2.

Few research works using biomarker-specific features in the classification of IHC slides have been reported so far. In the following, we give a brief comparison of these methods (Table 3). An extensive review and comparison of methods (not restricted to immunohistochemical quantification) used in the classification of breast cancer pathology slides is given in [15]. 

## 8. Conclusions and Future Work

This paper proposed a set of image features that are closely related to visual markers used for the HER2 classification of breast cancer histology slides. Specifically, four different types of features based on variations in stain intensity, texture characteristics, morphological variations and histogram statistics were considered. Characteristic curves represent the percentage of staining and its variation with saturation levels as a non-increasing smooth curve. Rotation-invariant uniform local binary pattern curves were used as texture descriptors. This paper also introduced a connectedness measure as a morphological feature of the staining patterns. The feature set was further augmented with global histogram features computed for the b* channel values of the image in the CIE-Lab colour space. Methods based on the geometrical characteristics of the features to visualize their distribution in the training set and also to reduce their dimensionality were presented. 

Further research work is directed towards analysing higher-order statistics of texture features including Grey Level Co-Occurrence Matrices (GLCMs) for improving the classification accuracy [29]. More texture features representing the morphological characteristics of membrane staining could help in reducing the overlap between regions corresponding to classes 1+ and 2+, and also between classes 2+ and 3+. It should also be noted that due to inaccuracies present in the process of IHC staining slides, there will always be some level of uncertainty in the stain intensity that will correspond to inaccuracies in the slide assessment [30]. The study of feature analysis will be followed by an extensive analysis of classification algorithms including neural networks, decision trees, random forests and more sophisticated deep learning algorithms.

## Figures and Tables

**Table 1:** Types and dimensions of features used in our analysis.

Features	Size	Key Properties
Characteristic curves	10	Non-increasing smooth curves
Rotation-invariant LBP	25	Very low curvature; similarity between curves
Connectedness	1	Large variations
Entropy, Energy	2	Inversely correlated

**Table 2:** Confusion matrix obtained from one trial run of the multi-class logistic regression algorithm.

		Predicted				Accuracy = 93.86%		
		0	1+	2+	3+	Precision	Recall	F1-Score
Actual	0	299	11	0	0	0.86	0.96	0.91
	1+	42	206	7	0	0.94	0.81	0.87
	2+	5	1	286	7	0.97	0.96	0.96
	3+	0	0	1	341	0.98	1.0	0.99

**Table 3:** A comparison of immunohistochemical (IHC) classification algorithms using biomarker-specific features. CNN: convolutional neural network.

Ref	Methods/Features	Classifiers	Accuracy
[14]	Colour pixel classifier Nuclei segmentation Ellipse fitting	Linear regression, Minimum cluster distance	80% Avg 86.5% Max
[18]	LBP Haralick features Histogram of oriented gradients	SVM CNN	80% Avg 92% Max
Our method	Characteristic curves ULBP Connectedness, Entropy	Logistic regression SVM	91% Avg 93% Max
